# Enhancing EEG-based attachment style prediction: unveiling the impact of feature domains

**DOI:** 10.3389/fpsyg.2024.1326791

**Published:** 2024-01-22

**Authors:** Ilan Laufer, Dor Mizrahi, Inon Zuckerman

**Affiliations:** Department of Industrial Engineering and Management, Ariel University, Ariel, Israel

**Keywords:** EEG data analysis, attachment styles, machine learning, feature domains, neurophysiological responses

## Abstract

**Introduction:**

Attachment styles are crucial in human relationships and have been explored through neurophysiological responses and EEG data analysis. This study investigates the potential of EEG data in predicting and differentiating secure and insecure attachment styles, contributing to the understanding of the neural basis of interpersonal dynamics.

**Methods:**

We engaged 27 participants in our study, employing an XGBoost classifier to analyze EEG data across various feature domains, including time-domain, complexity-based, and frequency-based attributes.

**Results:**

The study found significant differences in the precision of attachment style prediction: a high precision rate of 96.18% for predicting insecure attachment, and a lower precision of 55.34% for secure attachment. Balanced accuracy metrics indicated an overall model accuracy of approximately 84.14%, taking into account dataset imbalances.

**Discussion:**

These results highlight the challenges in using EEG patterns for attachment style prediction due to the complex nature of attachment insecurities. Individuals with heightened perceived insecurity predominantly aligned with the insecure attachment category, suggesting a link to their increased emotional reactivity and sensitivity to social cues. The study underscores the importance of time-domain features in prediction accuracy, followed by complexity-based features, while noting the lesser impact of frequency-based features. Our findings advance the understanding of the neural correlates of attachment and pave the way for future research, including expanding demographic diversity and integrating multimodal data to refine predictive models.

## 1 Introduction

Attachment styles offer profound insights into human behavior and neurophysiological responses, representing the intricate interplay between emotional and behavioral dynamics within close relationships (Vrtička and Vuilleumier, 2012; Sadikaj et al., 2015; Sheinbaum et al., 2015). Recent advances in electroencephalogram (EEG) research have unraveled the neural underpinnings of attachment styles. For instance, Verbeke et al. (2014) found that social contexts significantly influence cortical activity, particularly in individuals with anxious attachment styles, unveiling enhanced alpha, beta, and theta band activity in the presence of others, hinting at potential implications for social behavior and relationships. Simultaneously, Sloan et al. (2007) linked attachment anxiety with the presence of alpha waves during sleep, and Rognoni et al. (2008) unveiled associations between adult attachment styles and EEG frontal asymmetry, shedding light on emotional responses. Individuals with avoidant attachment exhibited reduced arousal and right frontal asymmetry to positive stimuli, while preoccupied individuals displayed heightened arousal and increased left frontal activation, indicating the profound impact of attachment experiences on emotions and neural activity.

In the context of ERPs, Ma et al. (2017) emphasized the role of early caregiver-infant interactions in shaping attachment styles and attention biases. Secure attachment correlated with accurate perception and response to infant signals, while anxiously attached individuals exhibited heightened P300 amplitudes when exposed to angry infant faces during a facial recognition task, revealing attachment style differences in brain responses to infant face perception. Additionally, Zuckerman et al. (2023a) highlighted how attachment style influences defensive responses, moderating the attachment system during the flanker task in the context of P200 and P400 ERPs.

Nonetheless, despite these valuable contributions, a conspicuous gap remains in the literature: the underexplored realm of AI models for predicting attachment styles based on EEG data. While attachment styles are fundamental to understanding human interaction, the field of emotion recognition, though closely related (Mikulincer and Shaver, 2005; Vrtička et al., 2012; Akhavan-Abiri et al., 2018; Zhang et al., 2023), has garnered more attention. Within emotion recognition, researchers have diligently explored a multitude of methodologies to harness EEG data for detecting emotional states and responses. These methodologies encompass comprehensive analyses of EEG signals, including time-domain and frequency-domain approaches, as well as advanced techniques like wavelet transforms, principal component analysis, and independent component analysis (Li et al., 2018; Alhalaseh and Alasasfeh, 2020; Liu et al., 2020; Jaswal and Dhingra, 2023; Vempati and Sharma, 2023). Moreover, recent studies have highlighted the potential of EEG-based emotion recognition, shedding light on its significance, particularly through the utilization of advanced techniques like Empirical Mode Decomposition (EMD), serving as an effective feature extraction method for capturing the complexity of emotional states from EEG signals (Zhuang et al., 2017; Mert and Akan, 2018; Wang and Wang, 2021).

While considerable advancements have been made in understanding emotional states through EEG data, the specific application of these techniques for predicting attachment styles, particularly through responses to cognitive tasks like the Flanker task (Eriksen and Eriksen, 1974), has not received the same level of attention. Addressing this research gap, our study aims to examine the potential of EEG responses to feedback in the Flanker task as distinguishing markers for secure and insecure attachment styles. Inspired by the work of Simon-Dack et al. (2021), who identified distinct EEG patterns associated with avoidance in a non-social context of the Flanker task, we seek to extend this inquiry. Our approach involves analyzing a variety of EEG features elicited by feedback in the Flanker task. This analysis is aimed at identifying EEG patterns that might distinguish secure from insecure attachment styles. We hypothesize that different attachment styles may manifest distinct EEG responses during the task, especially in relation to feedback processing, which could range from tendencies toward self-criticism to adaptability. By exploring these EEG patterns in the context of a cognitive task traditionally used for assessing performance monitoring (Overmeyer et al., 2023), our study endeavors to provide novel insights into the neural correlates of attachment styles.

We next turned our attention to the analytical methods employed in our study. Our research employs the XGBoost classifier for predicting attachment styles using EEG data. This methodological approach is notably scarce in attachment style literature, representing a significant utilization of the complexity inherent in EEG data for this specific context. The choice of XGBoost is based on its proven efficacy in managing the non-linear characteristics (Wang et al., 2021) inherent in EEG data, challenging for conventional analytical techniques (Chen and Guestrin, 2016) especially linear ones (Elgart et al., 2022). Our approach aligns with current trends in neuroscience and psychology, where ensemble machine-learning methods have shown effectiveness in interpreting complex neural patterns (Rahman et al., 2022; Li et al., 2023). Our study is focused on examining a broad range of EEG features, totaling 45 in number, which encompass elements from the time domain (Al-Fahoum and Al-Fraihat, 2014; Zuckerman et al., 2022, 2023b), frequency-based analyses (Mizrahi et al., 2022a,b, 2023a), and complexity measures (Sheehan et al., 2018; Ramadoss et al., 2022; Mizrahi et al., 2023b). The aim is to utilize these features to predict whether an individual has a secure or insecure attachment style and to assess the specific contribution of each feature to this prediction.

Building on our foundational work (Zuckerman et al., 2023b), our current study employs these diverse EEG features for a comprehensive analysis. This selection, encompassing a broad spectrum of domains, was chosen for its demonstrated significance in our previous research. In that study, most of the features, with a *p*-value of less than 0.0001, showed notable differences between secure and insecure attachment styles, except for three features. However, to ensure a comprehensive exploration of EEG patterns in attachment styles, all 45 features were included in the current analysis. Our present study adopts a predictive modeling approach, contrasting with the comparative focus of our earlier work (Mizrahi et al., 2023a; Zuckerman et al., 2023a,b). By using the XGBoost model, we aim to predict individual attachment styles based on the EEG features, moving beyond simple comparisons to a more nuanced understanding of the data. This methodological shift enhances our ability to accurately predict attachment styles and provides deeper insights into the neurophysiological patterns associated with these styles.

## 2 Methods

### 2.1 Data collection and participant recruitment

Our study was conducted in two phases. Initially, we recruited a group of 96 participants aged between 20 and 35 (mean = 24.25 years, SD = 2.0673), comprising 46 females, who were fourth-year engineering students. All participants were right-handed and reported no neurological symptoms. To assess their attachment styles, participants completed the ECR-R (Sibley and Liu, 2004; Sibley et al., 2005) questionnaire, a widely used self-report instrument consisting of 36 items grouped into anxiety and avoidance subscales. In our analysis of the ECR-R questionnaire results, we concentrated on two key dimensions: avoidance and anxiety, each scored on a scale from 1 to 7. In this scoring system, higher scores correspond to increased levels of insecurity within these dimensions. Participants characterized by lower scores were associated with secure attachment, while those with higher scores in both dimensions were indicative of fearful avoidant or disorganized attachment types. To avoid arbitrary thresholds for classifying these attachment types, we utilized the k-means clustering algorithm (Ahmed et al., 2020) to objectively determine natural groupings in our dataset. The k-means method effectively partitions data into clusters based on feature similarity. To determine the optimal number of clusters for k-means clustering, we employed the Elbow method, a widely-used heuristic in cluster analysis (Umargono et al., 2020). This method involves assessing the within-cluster sum of squares (WSS) against different numbers of clusters (k) (Grgic and Podobnik, 2021). WSS measures the compactness of clusters and decreases as k increases. The Elbow method identifies the point where increasing the number of clusters leads to diminishing returns in terms of decreasing WSS, typically signified by a noticeable ‘elbow’ in the trend. This ‘elbow’ point represents a balance between the number of clusters and the homogeneity within them. Our application of this method revealed *k* = 4 as the optimal number of clusters. Thus, our analysis, in line with established literature, indicated that four clusters – securely attached, anxiously attached, avoidant, and fearful avoidant/disorganized – were optimal. This classification, validated against existing demographic patterns (Magai et al., 2001), ensured the credibility of our methodology.

**Figure 1:**
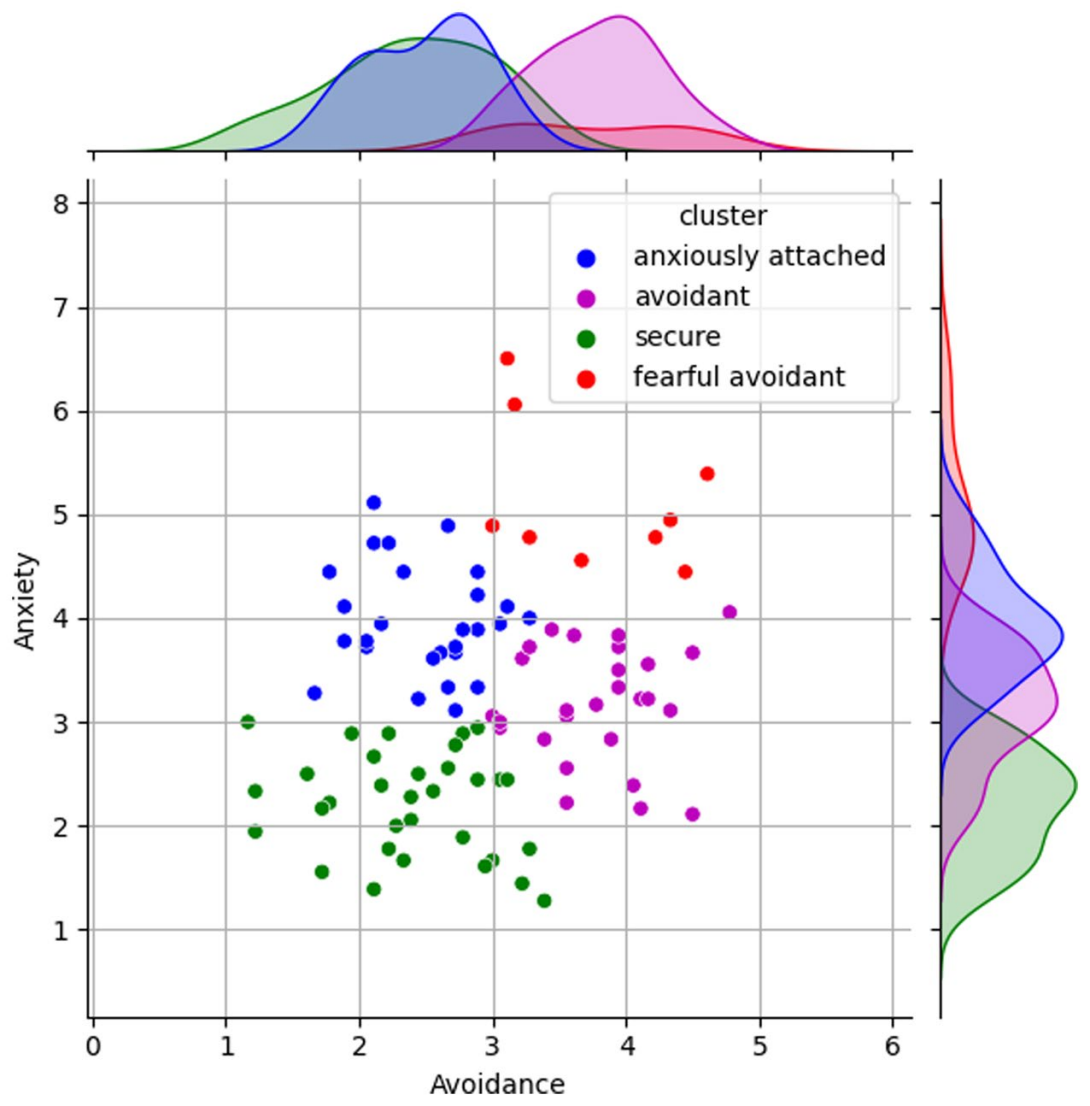
Grouped attachment outcomes based on the ECR-R questionnaire (*K* = 4).

For the EEG recording phase, 27 participants, representing the four identified attachment styles, were selected from an initial sample of 96 college students. The k-means algorithm (*k* = 4) classified these into a secure group of 6 and an insecure group of 21 (9 anxiously attached, 7 avoidant, 5 fearful avoidant) (Figure 1). Despite the resultant imbalanced cluster sizes, this distribution was necessary for ecological validity. By ensuring that the distribution of attachment styles in our sample closely resembled those found in broader, real-world populations, we aimed to enhance the applicability and relevance of our results. Such a representation, despite leading to unequal group sizes, was crucial in reflecting the actual prevalence and variation of attachment styles in a natural setting. We addressed potential impacts of this imbalance on our results, such as precision rates between groups, by using balanced accuracy (see Section 3.2) to evaluate our model’s performance accurately.

In our study, we strategically grouped attachment styles into two categories: secure and insecure. This simplification, based on our research’s focus, facilitated the initial application of machine learning methods, specifically XGBoost, to discern fundamental differences between these broad groups. This binary classification approach served as a crucial foundational step, enhancing the interpretability and statistical robustness of our findings. The decision to categorize attachment styles in this manner was instrumental in establishing a clear baseline for our explorations. It was a deliberate choice, aligning with our objective of utilizing machine learning to analyze attachment styles for the first time. Future research, building on this groundwork, will employ more sophisticated machine learning methods to explore the distinct features of each attachment style in greater depth, paving the way for a more thorough and detailed investigation.

During the EEG sessions, participants completed the flanker task (Brunetti et al., 2019). This task involved the rapid presentation of arrow flanker configurations, requiring participants to quickly identify the direction of a central arrow amidst distracting non-target arrows and respond using a keyboard. The primary objective of the flanker task was to evaluate how participants responded to the feedback provided for their task performance.

**Figure 2:**
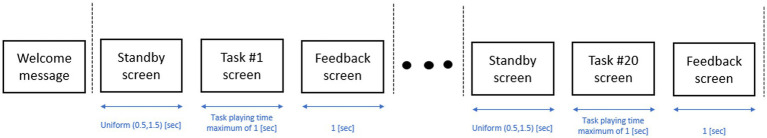
Experimental paradigm – single block.

Each participant performed 60 trials of the flanker task, divided into three blocks of 20 trials each, with a 1-min break between blocks (see Figure 2 in the experimental paradigm). Participants responded to on-screen arrows by pressing the corresponding arrow key. In the first and third blocks, they matched the direction of the target arrow, while in the second block, they pressed the opposite direction. Feedback was provided after each trial, with correct trials displayed in green as “correct” and incorrect trials in red as “incorrect” for 1 s. Between trials, a gray cross was displayed on a black screen, and participants focused on it for a randomly varying duration between 0.5 and 1.5 s. The duration of each trial was approximately 3 s, resulting in a total duration of approximately 60 s for each block comprising 20 tasks. Before the main task, participants underwent a training session to become acquainted with the experimental procedure.

### 2.2 EEG recordings

We recorded EEG signals using a 16-channel active EEG amplifier (e.g., USBAMP, by g.tec, Austria) operating at a sampling frequency of 512 Hz, adhering to the 10–20 international system operating at a sampling frequency of 512 Hz, following the 10–20 international system. Electrode impedance was maintained below 5 Kohm throughout the experiment, and data analysis focused on six frontal and prefrontal electrodes (Fp1, F7, Fp2, F8, F3, and F7). This selection of electrodes was guided by their established significance in cognitive neuroscience, especially for research on emotional processing (Zhang and Chen, 2020; Singh and Singh, 2021) and cognitive tasks (Zhang et al., 2020; Robble et al., 2021). The frontal and prefrontal regions are pivotal in regulating emotions and cognitive functions. These functions are essential to comprehend attachment styles and their impact on cognitive performance, a focus of our study where we employed the Flanker task.

### 2.3 EEG pre-processing

In our EEG data preprocessing, we utilized a bandpass filter within the 1–30 Hz range and a notch filter at 50 Hz to effectively minimize unwanted frequency components and noise. We focused on the Alpha, Beta, Theta, and Delta bands due to their established roles in emotional processing (Guo et al., 2022), attachment styles (Gander and Buchheim, 2015; Adenzato et al., 2019), and cognitive tasks such as the Flanker task (Vega et al., 2020; Clements et al., 2021). To enhance our signal quality, we employed Independent Component Analysis (ICA). ICA is a computational method used to separate a multivariate signal into additive, independent non-Gaussian components. This method is based on the assumption that the source signals are statistically independent and non-Gaussian. In the context of EEG data, ICA is particularly valuable for separating out artifacts (such as eye movements, muscle noise, or line noise) from the brain signals, since these artifacts are typically independent from the neural activity of interest (Pester and Ligges, 2018). By isolating these components, ICA allows for a clearer analysis of the underlying brain activity (Hyvärinen and Oja, 2000; Vigário et al., 2000).

The continuous filtered data were segmented into 1-s epochs, aligning with the flanker task slide duration. This approach allowed for the isolation and analysis of specific time intervals.

### 2.4 Classification

In our endeavor to differentiate between secure and insecure individuals based on EEG data, we employed a classification approach that relied on complexity-based, frequency-based, and time-domain-based features. After acquiring and preprocessing the EEG data, we extracted relevant features from the EEG epochs.

**Figure 3:**
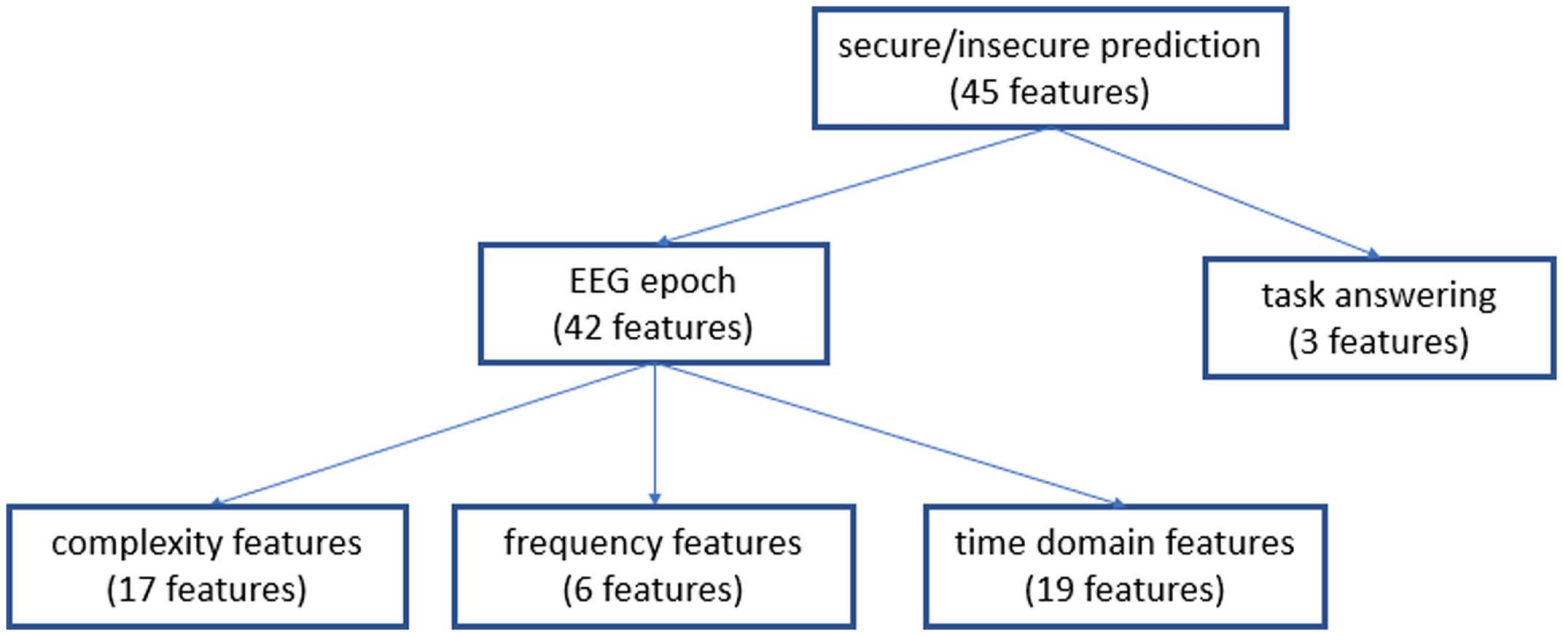
The distribution of the 45 features per EEG epoch, grouped into four categories.

These features were grouped into four categories. In total, 45 features were calculated for each EEG epoch (Figure 3) according to the following distribution:

1. Frequency-based features: This category included 6 attributes related to the relative values within the Alpha, Beta, Theta, and Delta frequency bands, as well as the ratios Theta to Alpha and Theta to Beta ratios, providing insights into the spectral characteristics of the EEG data.
2. Complexity-based features: This class contained 17 features designed to quantify the complexity and irregularity of EEG signals. Features such as Binned Entropy, Fourier Entropy, Lempel-Ziv Complexity, time series complexity, and sample entropy mean were computed across various bin sizes (2, 4, 8, 16, 32).
3. Time domain-based features: Comprising a total of 19 distinct attributes, this category captured temporal characteristics and patterns in the EEG data.
4. Trial feedback dynamics: This category comprised 3 features including response time and the success/failure feedback valence, both in the current and previous trials, which are all essential aspects of the feedback process.

For a comprehensive list of these 45 EEG-based features, please refer to Table A1. For detailed definitions of the EEG features used in our study, such as ‘count above mean/median’, ‘first max location’, ‘min location’, etc., please refer to the tsfresh documentation, available at: https://tsfresh.readthedocs.io/en/latest/text/list_of_features.html.

In our study, we calculated each feature across six frontal electrodes and used their average values to enhance the robustness of our results. The ensemble model, combining these models from the frontal electrodes, demonstrated superior classification performance on the test set. Our dataset included 1,240 samples of the insecure attachment type and 360 samples of the secure type. Employing k-fold cross-validation, we partitioned our dataset of 1,600 epochs into four folds (1,280 epochs) for training, with one fold (320 epochs) reserved for testing in each iteration. This approach utilized the XGBoost algorithm, known for its effectiveness in classification tasks (Wong, 2015; Sun, 2020).

To address the challenges inherent in small sample sizes for predictive modeling, our study utilized K-fold cross-validation on a 1,600-epoch dataset. This approach helps reduce bias, which is especially beneficial as our sample size goes beyond the 1,000-epoch benchmark set for small samples in K-fold validation (Vabalas et al., 2019). Similarly, a study on the DEAP dataset (Koelstra et al., 2011) employing Gradient Boosting Machines (GBMs) for emotion classification used a leave-one-participant-out cross-validation method with a total sample size of 1,280 (32 participants × 40 samples each) (Aggarwal et al., 2018). This research highlighted the effectiveness of GBMs in small datasets, achieving high accuracy with optimized features and parameters, and providing supportive evidence for strong model performance in settings with limited samples. Additionally, research exploring the influence of sample size on predictive algorithms for psychological treatment prognosis indicated that decision-tree ensemble models, similar to those we utilized, begin to achieve optimal performance at sample sizes of approximately 1,000 to 2,000 for AUC (McNamara et al., 2022).

In summary, we utilized a mix of complexity-based, frequency-based, and time-domain-based features extracted from the EEG epochs. By integrating k-fold cross-validation with the XGBoost algorithm, our model aims to effectively classify between secure and insecure attachment styles based on EEG data, aligning with recent trends in EEG-based machine learning research (Tiwari and Chaturvedi, 2019).

## 3 Results

### 3.1 Precision disparities in attachment prediction

Our investigation revealed notable differences in prediction performance between secure and insecure attachment styles, as depicted in Table 1. Our analysis of EEG features, which included time-domain, complexity-based, and frequency-based attributes, using the XGBoost classifier, unveiled significant insights into our predictive model’s accuracy. In our analysis, we found distinct patterns in the precision of attachment style classification (see Table 1). Notably, the XGBoost classifier exhibited a high precision of 96.18% in identifying individuals with insecure attachment styles, reflecting its ability to accurately recognize those with heightened anxiety and avoidance tendencies. In contrast, achieving precision in secure attachment classification proved to be more challenging, with a precision rate of 55.34%. This disparity underscores the complexity of distinguishing individuals with secure attachment styles within the dataset. Furthermore, the confusion table data reveal a False Discovery Rate (FDR) for secure attachment classification of 44.66%. This indicates that while the model may occasionally misclassify individuals as having a secure attachment style, the majority of these instances actually represent individuals with insecure attachment styles. Conversely, the FDR for insecure attachment classification is notably lower, at 3.82%, demonstrating a lower likelihood of false positive identifications.

**Table 1:** Confusion matrix for attachment style prediction.

		Predicted classes		True positive rate	False negative rate
		Insecure *p* = 0	Secure *p* = 1		
True classes	Insecure *p* = 0	981	259	79.11%	20.89%
	Secure *p* = 1	39	321	89.17%	10.83%
Positive predicted value		96.18%	55.34%	Prediction accuracy 81.37%	
False discovery rate		3.82%	44.66%		

In conclusion, we reached a recall of 80% for Insecure prediction and about 90% for the Secure prediction. The unbalanced nature of the dataset, with a large number of errors in the Insecure group (259), impacted the model’s prediction accuracy for the Secure group, making it approximately 55% as opposed to around 96% for the Insecure group. This indicates that predictions from the Insecure group are more reliable than those from the Secure group in the context of this dataset. Overall, our predictive model demonstrates an 81.37% prediction accuracy.

### 3.2 Balanced accuracy and precision enhancement evaluation

To accurately assess the efficacy of our attachment prediction model, we utilized the balanced accuracy metric. This metric is a standard in classification tasks, as it incorporates both sensitivity (True Positive Rate) and specificity (True Negative Rate). Such an evaluation method is particularly important for datasets with an imbalance, such as ours (see Equation 1).


(1) $$\begin{array}{l} \mathrm{Balanced}\;\mathrm{accuracy}=0.5*\left(\frac{TP}{TP+FN}+\frac{TN}{TN+FP}\right)\\ \;=0.5*\left(\frac{321}{321+39}+\frac{981}{981+259}\right)=84.14\% \end{array}$$

We determined a balanced accuracy of approximately 84.14%. This demonstrates an enhanced precision in categorizing individuals based on their attachment styles while taking into account the dataset’s inherent skew.

Sensitivity evaluates the model’s effectiveness in correctly identifying those with secure attachment tendencies, while specificity assesses its ability to exclude those without these tendencies. Initially, the model reported a balanced accuracy of about 81%, indicating its performance before addressing the dataset’s imbalance. After making adjustments to enhance precision, the model’s balanced accuracy increased to approximately 84%. This improvement signifies the model’s refined capability to classify individuals based on their attachment styles, considering the challenges presented by the dataset imbalance.

### 3.3 Model accuracy across attachment style spectrum

Attachment styles, being multifaceted, more closely resemble a spectrum than fixed categories. This spectrum is visually represented on a two-dimensional plane, with the axes symbolizing anxiety and avoidance — the key determinants of attachment styles. In essence, while the model demonstrates proficiency in identifying pronounced insecure tendencies, its performance becomes more variable when tasked with discerning subtler nuances, particularly in regions where secure and insecure characteristics aren’t starkly distinct. This nuance, and occasionally the model’s inability to perfectly navigate it, leads to periodic misclassifications. The origin of two-dimensional plane serves as a balanced point for both elements. Using the Equation 2:


(2) $$R_{\mathrm{attachment}}=\sqrt{{\left(\mathrm{avoidance}\right)}^{2}+{\left(\mathrm{anxiety}\right)}^{2}}$$

We can gauge the distance of a player from the origin, which serves as an indicator of their attachment style.

#### 3.3.1 Secure group

**Figure 4:**
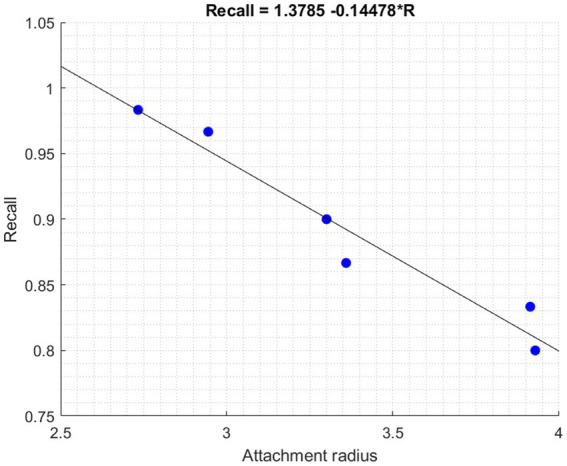
Relationship between attachment radius and recall for the secure group.

Those closer to the origin predominantly exhibit secure tendencies. Imagine a tight circle around the origin: a smaller radius within this circle represents those with pronounced secure attachments. However, as we move outwards, increasing the radius, the strength of these secure tendencies begins to diminish. Our analysis dove deeper into understanding the model’s proficiency by assessing the recall value for Secure players based on their attachment radius (Figure 4).

The relationship between recall and radius is captured by the equation: Recall = 1.3785–0.14478 × *R*. A negative relationship is evident: as the radius *R* increases, moving toward the Insecure group, the recall value decreases, indicating reduced classification accuracy. Conversely, for those closer to the origin with a smaller *R* (indicating stronger secure tendencies), the model performs with heightened accuracy.

#### 3.3.2 Insecure group

**Figure 5:**
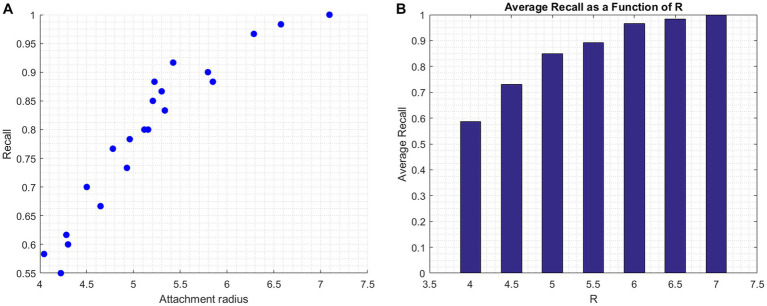
Relationship between attachment radius and model recall. **(A)** (Scatter plot): Displays the correlation between individual participants’ attachment radius and recall values. Each dot signifies a participant from the insecure group, mapping their radius to the model’s classification accuracy. **(B)** (Bar graph): Represents the average recall values for participants based on specific radius ranges.

Individuals situated further away from the origin, distinguished by a larger radius, predominantly display insecure tendencies. These are individuals who often exhibit higher levels of either anxiety, avoidance, or both. Think of a broader circle on our plane; its expansive nature encapsulates the varied manifestations of insecurity. In Figure 5, we explore the nuances of attachment tendencies through a visual representation of the model’s recall accuracy in relation to the attachment radius. In Figure 5A, each dot represents an individual participant from the insecure group This scatter plot reveals the correlation between attachment radius and recall values, emphasizing that individuals with a larger radius — further away from the origin — are more likely to be accurately identified by the model as having insecure tendencies.

Figure 5B displays bars representing the average recall value for participants within specific radius brackets. It can be seen that the bars gradually increase in size with an increasing radius, however, this increase becomes more subtle and less pronounced at higher radii. Integrating our understanding from the figures, individuals situated further from the origin, marked by a larger radius, predominantly exhibit insecure tendencies. These are individuals who often manifest higher levels of either anxiety, avoidance, or both. The broader circle on our plane captures the diverse shades of insecurity.

### 3.4 Contribution of EEG feature domains to attachment style classification

**Figure 6:**
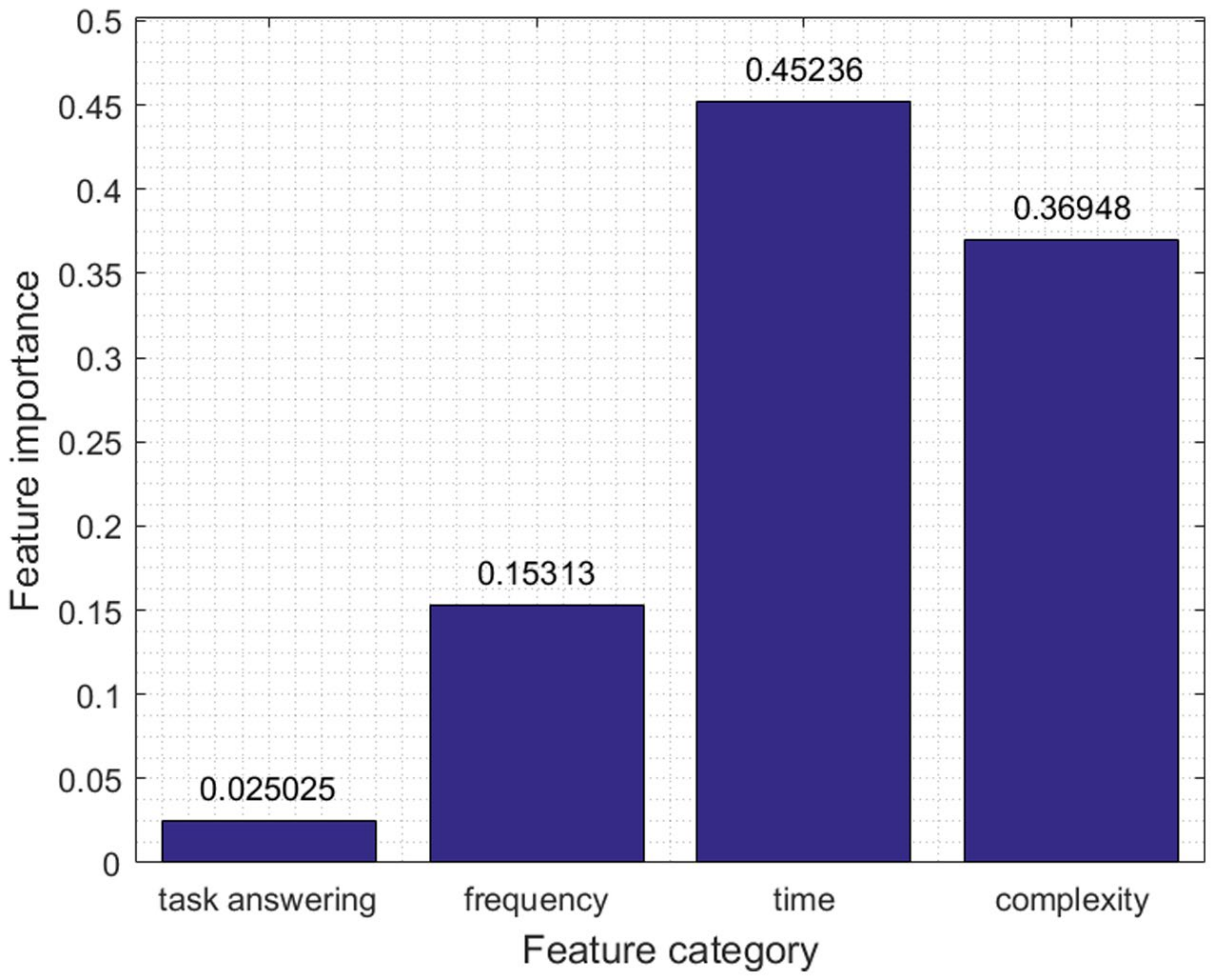
EEG feature importance in attachment prediction.

The success of the classification model in differentiating attachment styles is rooted in the informative strength of various EEG feature domains. As visualized in Figure 6, where the y-axis denotes feature importance, we have extracted and analyzed features across distinct classes (x-axis), each contributing differently to the predictive accuracy.

Specifically, we explored the distinctive contributions of various EEG feature domains to the classification of attachment styles. The contribution of each kind of feature in our study was determined using XGBoost’s inherent ability to assess feature importance. XGBoost automatically evaluates the relevance of each feature within the predictive model it constructs. Upon building the boosting trees, the algorithm assigns an importance score to each feature, reflecting its utility in constructing the model’s decision trees. These scores are based on criteria such as the frequency of a feature’s usage in splits, the average gain of model accuracy from a feature, and the coverage of the feature across the data. Consequently, features that are more frequently used in pivotal splits leading to significant improvements in model accuracy receive higher importance scores. This feature importance score, therefore, serves as a quantitative measure of each feature’s contribution to the model’s predictions, offering an objective and automated insight into the predictive dynamics of the model (Chen and Guestrin, 2016). Notably, the predictive accuracy of our model was influenced by the informative strength of each feature domain. Frequency-based features, encompassing relative values within EEG bands such as Alpha, Beta, Theta, and Delta, including Theta-to-Alpha and Theta-to-Beta ratios, made a moderate contribution of 15% to the overall prediction accuracy. In contrast, Complexity-Based Features, quantifying EEG signal complexity and irregularity through measures like Binned Entropy, Fourier Entropy, Lempel-Ziv Complexity, time series complexity, and mean sample entropy, played a more prominent role, contributing significantly with 37%. Time Domain-Based Features emerged as the top contributors, accounting for a substantial 45% of the overall prediction accuracy, capturing temporal patterns and characteristics within the EEG data.

Conversely, Trial Feedback Dynamics, which included the factors of response time and feedback valence, had the least impact, contributing a mere 2% to the prediction accuracy. These findings underscore the importance of considering distinct EEG feature domains when developing predictive models for attachment styles, with temporal characteristics being particularly influential.

### 3.5 Model performance and implications of attachment radius

In analyzing the model’s performance, several nuances emerge that have influenced its predictive accuracy. One significant observation is the model’s inclination to misclassify a notable fraction (20.89%) of the Insecure group as Secure. This misclassification can be linked to those with a low attachment radius, specifically within the [4, 5.5] range, as evident in Figure 5A. Their closeness to the Secure group, due to the low radius, poses inherent classification challenges. However, the distinction between groups becomes clearer with increasing radius values.

The recall metrics provide further insight: the Secure group correctly identified its members with an accuracy of 89.17%, whereas the Insecure group exhibited a recall of 79.11%. Precision metrics reveal a stark contrast; the Insecure group’s Positive Predicted Value (PPV) was 96.18%, whereas the Secure group’s PPV stood at only 55.34%. This points to the greater susceptibility of the Secure group to misclassification, further evidenced by their higher False Discovery Rate of 44.66%, compared to the Insecure group’s 3.82%. In summation, the model achieved an overall prediction accuracy of 81.37%.

The imbalanced nature of our training dataset, with 21 insecure players to 6 secure players, accentuates the impact of these misclassifications. Out of 1,240 epochs, 259 were errors, which has tangible implications for the precision metrics of the Secure group. Nevertheless, the model’s ability to discern between attachment styles, even amidst dataset imbalance, underscores its adaptability and real-world applicability. The fact that it aligns its predictions in tune with the radius — a derived feature not directly fed into the model — indicates its capacity for genuine learning rather than mere pattern recognition or memorization.

## 4 Discussion

Building on recent neuroscientific advances, our primary aim was to determine the potential of EEG data in predicting and differentiating between secure and insecure attachment styles. This exploration sought not only to enhance the precision of such predictions but also to understand which EEG feature domains are most influential in the context of attachment styles. The use of EEG data to examine attachment styles offers a novel perspective into the neural correlates of interpersonal dynamics (White et al., 2023). Our findings delineate various facets: the precision differences in predicting attachment styles, the relationship between the degree of perceived insecurity and prediction accuracy, and the significance of specific feature domains.

In our study, while acknowledging the existing dimensional conceptualization of attachment styles, we introduce the ‘attachment radius’ as a novel metric to quantify these dimensions. This concept is inspired by the evolution in Social Value Orientation (SVO) measurement (Murphy et al., 2011), reflecting a similar shift from categorical to continuous assessment. Our ‘attachment radius’ offers a quantifiable representation of attachment styles, encapsulating their dimensionality in a measurable format. This methodological innovation allows for a more detailed and precise analysis of attachment styles, paralleling advancements in psychological constructs. The theoretical and practical implications of this approach are further explored in Sections 4.1 and 4.2.

### 4.1 Precision discrepancies in attachment style prediction

One of the salient observations from our research was the precision rate discrepancy between predictions for secure and insecure attachment styles. With a 96% precision rate for insecure predictions versus a 55% for secure ones, the data highlights the intricate nature of leveraging EEG patterns for these predictions. Further analysis revealed occasional misclassifications, particularly of insecurely attached individuals as securely attached. This can be partly attributed to the complexity of attachment styles existing on a spectrum. Individuals within the insecure attachment spectrum who exhibit traits not strongly indicative of either anxious or avoidant styles were more prone to misclassifications. Similarly, while the neural responses of individuals with pronounced insecure attachment characteristics are varied and distinct, making them generally more identifiable, those with subtler insecure traits present challenges in accurate classification. Conversely, securely attached individuals often demonstrate less distinct neural patterns, contributing to the lower precision rate in identifying secure attachment. This detailed understanding of the attachment spectrum and the variability of neural responses highlights the challenges of using EEG data for attachment style predictions. Such nuances emphasize the challenges and the necessity for refinement when using EEG data for predictions on attachment styles (Yadav et al., 2018; Rahman et al., 2021).

To understand the reasons behind the precision rates of our model (96% for the insecure group vs. 55% for the secure group), we identified several key factors influencing this outcome, beyond the implications of the imbalanced sample size. Firstly, according to the literature, the neural responses of individuals with insecure attachment styles are more varied and pronounced (Verbeke et al., 2014; Gander and Buchheim, 2015; Zuckerman et al., 2023a), making them more easily identifiable by the model. In contrast, the neural patterns of securely attached individuals are less distinct (Zuckerman et al., 2023a), leading to lower precision in their identification. Furthermore, the spectrum nature of attachment styles complicates the classification task, particularly when discerning subtle nuances between secure and insecure traits. Additionally, the differential impact of EEG feature domains on the model’s precision is closely tied to the imbalance in precision rates between insecure and secure groups. For instance, Time Domain-Based Features, which significantly contribute to model precision, may be more effectively capturing the variability in neural patterns associated with insecure attachment styles. This leads to a higher precision rate in identifying insecure attachments. In contrast, securely attached individuals, whose neural patterns might not be as distinctly captured by these dominant features, are more prone to misclassification, contributing to the lower precision rate. Furthermore, the subtler influence of Trial Feedback Dynamics suggests that these features might not sufficiently differentiate between secure and insecure attachment styles, further contributing to the imbalance in precision rates. This indicates a need to refine the feature selection or model tuning specifically for the secure group to balance the precision rates.

### 4.2 Neural pattern distinctiveness and recall efficacy

In our EEG study focused on predicting attachment styles, we examined the impact of $R_{\mathrm{attachment}}$ size on recall magnitude, revealing differential behavior among individuals with secure and insecure attachments. This critical observation directly informs the contrasting trends observed in Figures 4, 5, which relate to $R_{\mathrm{attachment}}$ , a metric used to measure attachment style proximity to a secure baseline in a two-dimensional avoidance-anxiety framework. Smaller $R_{\mathrm{attachment}}$ values signify closer alignment with secure attachment (lower avoidance and anxiety), while larger values denote insecure attachment.

The negative correlation between $R_{\mathrm{attachment}}$ and recall among securely attached individuals in our study aligns with the phenomenon of reduced distinctiveness or within-group variance in neural patterns, a pattern suggested by prior literature (Zuckerman et al., 2023a). This pattern can be attributed to their relatively consistent attachment behaviors, resulting in more uniform neural patterns when $R_{\mathrm{attachment}}$ is smaller, indicating a closer alignment with secure attachment (characterized by lower avoidance and anxiety). This uniformity in neural patterns enhances our EEG-based model’s ability to detect deviations from the normative pattern, including subtle ones, ultimately leading to higher recall rates. Conversely, in the insecure attachment group, as $R_{\mathrm{attachment}}$ values increase, individuals tend to exhibit higher levels of insecurity. As attachment insecurity grows, according to the literature, neural patterns tend to become more diverse and less uniform (Verbeke et al., 2014; Gander and Buchheim, 2015; Zuckerman et al., 2023a), reflecting the broader spectrum of behaviors and experiences associated with insecure attachment. Within this context, our EEG-based model benefits from larger deviations (indicated by higher $R_{\mathrm{attachment}}$ values), as they enhance the discernibility of these more diverse patterns, making model classification and recall easier.

The ‘gray zone,’ which falls approximately between $R_{\mathrm{attachment}}$ values of four to five, is worth noting for its unique characteristics (see Figure 5). Within this range, neural patterns of secure and insecure attachment styles begin to overlap, presenting challenges for accurate model classification. The marked differences in neural pattern discernibility observed at the extremes of the $R_{\mathrm{attachment}}$ spectrum become less pronounced in this region. For instance, Fraedrich et al. (2010) found minimal EEG differences between secure and certain insecure mothers within this gray zone, illustrating the difficulty of differentiation based solely on EEG patterns. In the gray zone, recall accuracy may decrease for securely attached individuals, as their typically less noticeable neural patterns begin to resemble those associated with insecure attachment variability. Conversely, for the insecure attachment group, this is where their usually more varied neural patterns become less distinguishable from those of securely attached individuals, posing classification challenges. The model’s recall performance in the gray zone becomes particularly challenging because the distinctiveness of the neural patterns diminishes for both securely and insecurely attached individuals, making it difficult for the model to make accurate classifications based on EEG data alone. Thus, the gray zone represents a unique and complex region where the model’s performance faces significant challenges, and additional information or features may be necessary for improved classification.

The model’s recall performance depends on deviations from the normative pattern. Securely attached individuals typically exhibit uniform neural patterns due to their consistent attachment behaviors. While this uniformity aids in detecting subtle deviations, it can also lead to challenges in predicting attachment styles. The model’s heightened sensitivity to small variations, less meaningful in secure attachment, can result in overclassification. Conversely, the model excels at recognizing diverse neural patterns seen in insecure attachment. Insecure attachment typically involves a broader range of behaviors, resulting in more varied neural patterns among individuals. Larger deviations (higher $R_{\mathrm{attachment}}$ values) enhance distinctiveness, aligning with the XGBoost model’s proficiency in identifying patterns with noticeable variability or differences (Tiwari and Chaturvedi, 2019).

In developing the concept of ‘attachment radius,’ we drew inspiration from advancements in SVO measurement (Murphy et al., 2011). While the dimensional conceptualization of attachment styles is established, our study introduces a new metric to quantify this dimensionality. SVO is a psychological construct that describes how people make decisions in social situations, particularly regarding the allocation of resources. It measures individuals’ preferences when distributing resources between themselves and others, indicating their level of cooperativeness, selfishness, or competitiveness. The SVO Slider method, a tool for assessing SVO, uses angles to continuously represent these social preferences, offering a detailed view of an individual’s orientation toward social cooperation or self-interest. Our ‘attachment radius’ concept similarly translates the complex, multidimensional nature of attachment styles into a measurable, continuous spectrum, akin to the SVO Slider’s approach (Murphy et al., 2011).

This innovation mirrors the evolution in SVO measurement and adds a new layer of precision to the understanding of attachment styles, offering a fresh perspective in attachment theory research. While the ‘attachment radius’ in our current study was applied to analyze collective patterns within attachment style groups, its potential extends beyond this initial application. In future research, this metric could be refined to assess individual variability in attachment styles. Such an advancement would enable a more personalized analysis, providing insights into each person’s unique position on the spectrum of attachment. This prospective development promises to transform the ‘attachment radius’ from a tool for group-level analysis to a more precise instrument for individual assessment.

### 4.3 Feature domain significance

A focal point of our research was understanding the contributions of different feature domains to attachment style prediction. The results highlighted the time domain’s pivotal role in prediction accuracy, accounting for 49% of the contribution. This underscores the importance of temporal dynamics in EEG data when investigating psychological constructs such as attachment styles (Kim and Seo, 2003; Kang et al., 2016; Uyulan and Erguzel, 2017; Ibagon, 2018). Complexity features, too, proved influential, contributing 36% to the predictive accuracy. This fact emphasizes that intricate EEG patterns can hold significant information for analyses in the realm of psychology (Lau et al., 2022; Lord and Allen, 2023). On the other hand, the contribution of frequency features was more modest, accounting for a 15% share in prediction accuracy. This observation suggests that while frequency dynamics in EEG data can be informative, they might not be the primary indicators for differentiating attachment styles (Bekkedal et al., 2011; Du et al., 2020).

Features related to Trial-feedback Dynamics, especially those associated with participant feedback, exhibited minimal influence on attachment style prediction. This infers that inherent neural patterns might be more indicative of attachment styles than task-induced responses during EEG sessions. However, at this stage this is still speculative and while there is no direct comparison of the weight of intrinsic and task-related EEG factors, it is generally known that both types of factors can impact cognitive processes and task performance (Karamacoska et al., 2018; Mueller et al., 2021; Cross et al., 2022).

### 4.4 Limitations and future studies

Our sample’s composition, predominantly students, poses a limitation in representing the diverse demographic variables influencing attachment styles. To partially address this, we applied a proportional allocation method and k-means clustering, which yielded a distribution aligning with the four attachment groups recognized in literature (Magai et al., 2001). This approach, however, does not fully overcome the inherent biases from the initial participant pool. Future research should strive for a broader demographic representation, including variations in age, socioeconomic status, and cultural backgrounds, and explore the dynamic evolution of attachment styles through longitudinal studies.

In addition to broadening the demographic diversity of future studies, there is also room for methodological advancements in our approach. The methodological approach employed here, which utilized k-fold cross-validation with the XGBoost algorithm, provides a foundation for further refinement and exploration. Investigating alternative classification algorithms or ensemble methods may offer avenues to enhance predictive accuracy. With attachment styles being dynamic, a time series analysis could provide insights into EEG patterns’ temporal evolution linked to attachment shifts (e.g., Li et al., 2018). Building on the EEG analysis, integrating data from additional modalities such as fMRI or hormone levels (e.g., Coan and Allen, 2004) can deepen insights into EEG correlates of attachment and improve the robustness and generalizability of predictive models.

Expanding our research methodology to include diverse data sources sets the stage for exploring novel clinical applications. One such application is understanding the ‘attachment radius’ alongside ERP components and psychophysiological assessments, which opens new avenues in the clinical diagnosis and treatment of attachment-related disorders. Our study specifically paves the way to identify individuals with a larger ‘attachment radius’ and subsequently assign them to specific tailored treatments. For individuals with a larger ‘attachment radius’, indicative of a more diverse range of neurophysiological responses typically seen in insecure attachment styles (Cuoco et al., 2021) targeted interventions could be designed to address their specific emotional and psychological needs. These interventions might include strategies aimed at regulating emotional reactivity, enhancing interpersonal relationships, or developing coping mechanisms for stress and anxiety. Furthermore, the ‘attachment radius’ metric can serve as a crucial tool in monitoring the effectiveness of therapy. By tracking changes in the ‘attachment radius’ over the course of treatment, clinicians can gauge the individual’s progress and adjust therapeutic strategies accordingly. This approach holds promise for more precise diagnostics and personalized medicine, offering a new paradigm in the management and treatment of attachment-related psychological conditions.

As we explore the clinical applications of the ‘attachment radius,’ the underlying data analysis techniques become increasingly crucial. In this study, we employed XGBoost for analyzing EEG data. Necessary preprocessing steps were undertaken to transform raw EEG signals into a structured form amenable for analysis with XGBoost. It is important to note that while these preprocessing steps make the EEG data more suitable for analysis with XGBoost, they might also alter the original signal characteristics to some extent. Therefore, future research should explore the balance between preprocessing EEG data for machine learning applications and preserving the integrity of the original signal.

In comparison, deep learning models, especially those tailored for time-series data, are often posited as more naturally fitting for raw EEG data (Roy et al., 2019). Models like Convolutional Neural Networks and Recurrent Neural Networks have the capability to automatically extract features from high-dimensional data, potentially allowing for a deeper understanding of EEG signals without extensive preprocessing. However, these models typically require larger datasets and substantial computational resources. While deep learning models offer powerful capabilities for raw data analysis, XGBoost presents a more accessible option in scenarios with limited computational resources or smaller datasets.

Future research should aim to compare the efficacy of XGBoost and deep learning approaches in EEG data analysis. This would help in elucidating the strengths and limitations of each method, guiding researchers toward the most suitable approach for their specific EEG data analysis requirements.

### 4.5 Concluding remarks

Our study highlights the utility of EEG data in identifying neural patterns associated with attachment styles. The prominence of time and complexity domains in attachment prediction showcases the depth and challenges of analyzing EEG signals. The variation in prediction precision between secure and insecure attachments also suggests the range of challenges associated with classifying these styles. Specifically, the association between the degree of perceived insecurity and prediction accuracy highlights the challenges in deciphering EEG patterns related to attachment styles. This research serves as a step toward understanding the neural correlates of attachment, prompting further exploration in the domain (see Vempati and Sharma, 2023).

## Data availability statement

The raw data supporting the conclusions of this article will be made available by the authors, without undue reservation.

## Ethics statement

The studies involving humans were approved by Ethic Committee of Ariel university (confirmation number: AU-ENG-IZ-20220404). The studies were conducted in accordance with the local legislation and institutional requirements. The participants provided their written informed consent to participate in this study. Written informed consent was obtained from the individual(s) for the publication of any potentially identifiable images or data included in this article.

## Author contributions

IL: Conceptualization, Data curation, Formal analysis, Investigation, Methodology, Supervision, Validation, Visualization, Writing – original draft, Writing – review & editing. DM: Conceptualization, Data curation, Investigation, Methodology, Software, Supervision, Validation, Visualization, Writing – original draft, Writing – review & editing. IZ: Conceptualization, Data curation, Formal analysis, Investigation, Methodology, Supervision, Validation, Visualization, Writing – original draft, Writing – review & editing.

## Appendix A: Categorization of the EEG features.

TABLE A1 Complete list of the 45 EEG features employed in the study.

**Table:**

Time-domain features	Complexity features	Frequency-based features	Trial-feedback dynamics
Mean absolute energy Max amplitude Sum of absolute changes Count above mean Count above median First max location First Min Location Kurtosis Last Max Location Last min location Longest strike above mean Longest strike above median Mean absolute change Mean change Number of crossing mean Number of crossing median Range count 25% to 75% Skewness Variation coefficient	Binned entropy (2, 4, 8, 16, 32) Fourier entropy (2, 4, 8, 16, 32) LZC (2, 4, 8, 16, 32) cid_ce Sample entropy	Relative Delta power Relative Theta power Relative Alpha power Relative Beta power Theta to Alpha ratio (TAR) Theta to Beta ratio (TBR)	Response time (ms) Feedback valence (current Trial) Feedback valence (previous trial)
